# Evolutionary Cell Biology of Division Mode in the Bacterial *Planctomycetes*-*Verrucomicrobia*- *Chlamydiae* Superphylum

**DOI:** 10.3389/fmicb.2016.01964

**Published:** 2016-12-06

**Authors:** Elena Rivas-Marín, Inés Canosa, Damien P. Devos

**Affiliations:** Centro Andaluz de Biología del Desarrollo, Consejo Superior de Investigaciones Científicas, Junta de Andalucía, Universidad Pablo de OlavideSeville, Spain

**Keywords:** PVC superphylum, peptidoglycan, cell division, budding, ftsZ, *dcw* cluster

## Abstract

Bacteria from the *Planctomycetes*, *Verrucomicrobia*, and *Chlamydiae* (PVC) superphylum are exceptions to the otherwise dominant mode of division by binary fission, which is based on the interaction between the FtsZ protein and the peptidoglycan (PG) biosynthesis machinery. Some PVC bacteria are deprived of the FtsZ protein and were also thought to lack PG. How these bacteria divide is still one of the major mysteries of microbiology. The presence of PG has recently been revealed in *Planctomycetes* and *Chlamydiae*, and proteins related to PG synthesis have been shown to be implicated in the division process in *Chlamydiae*, providing important insights into PVC mechanisms of division. Here, we review the historical lack of observation of PG in PVC bacteria, its recent detection in two phyla and its involvement in chlamydial cell division. Based on the detection of PG-related proteins in PVC proteomes, we consider the possible evolution of the diverse division mechanisms in these bacteria. We conclude by summarizing what is known and what remains to be understood about the evolutionary cell biology of PVC division modes.

## Introduction

**FIGURE 1:**
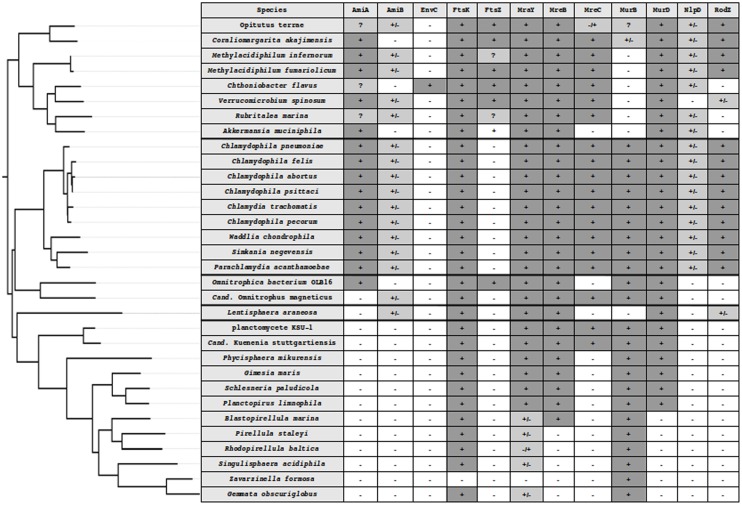
**Phylogenetic tree of the PVC superphylum and detection of division and PG synthesis-related proteins in PVC.** PVC species from five well-supported monophyletic groups, *Verrucomicrobia*, *Lentisphaera*, *Chlamydiae*, *Planctomycetes*, and *Cand*. Omnitrophica. The tree was built using 16S rRNA sequences in PhyML 3.1 with custom parameters and the HKY85 matrix (Guindon et al., 2010) and rooted on *E. coli*. *In silico* protein detection in the PVC proteomes is denoted by the following symbols: -, not found; +, found from both searches; +/-, found in the search starting from the *W. chondrophila* sequence but not from the one starting from *E. coli*; -/+, found in the search starting from the *E. coli* sequence but not from the one starting from *W. chondrophila*; ?, indicating that a candidate could be found using an alternative query (as in the case of *Methylacidiphilum infernorum* FtsZ), that only a fragment of the protein could be found (incomplete data, as in the case of *Rubritalea marina* FtsZ) or that both searches found different proteins that were the reciprocal best hit of both queries (as in the case of AmiA in *Verrucomicrobia*).

Most bacteria divide by binary fission using a mechanism centered on the interaction between the FtsZ protein and the peptidoglycan (PG) biosynthesis machinery. With limited exceptions, both FtsZ and PG are ubiquitous in bacteria. Amongst those exceptions are the members of the *Planctomycetes*, *Verrucomicrobia*, and *Chlamydiae* (PVC) superphylum, which encompasses these three phyla as well as the *Lentisphaerae* and some uncultured candidate phyla, such as the *Candidatus* Omnitrophica (previously known as OP3; Wagner and Horn, 2006; Fuerst, 2013a; Devos and Ward, 2014) (**Figure 1**).

The *Chlamydiae* phylum was initially restricted to members of the family *Chlamydiaceae* due to their human pathogenicity, but now includes the families *Parachlamydiaceae, Waddliaceae, Criblamydiaceae*, and *Simkaniaceae* of the order *Chlamydiales* (Bertelli and Greub, 2013). These newly described families of chlamydia-related bacteria diverged from the *Chlamydiaceae* more than 700 million years ago (Horn et al., 2004). The phyla *Lentisphaerae* and *Verrucomicrobia* form a separate group within the PVC superphylum, with the latter containing the orders *Opitutales*, *Puniceicoccales*, and *Verrucomicrobiales*, amongst others. Within the *Planctomycetes*, three orders are recognized: *Cand.* Brocadiales, *Phycisphaerales*, and *Planctomycetales*. The *Cand.* Brocadiales is a deep-branching order responsible for anaerobic ammonium oxidation (anammox; van Niftrik, 2013). The *Phycisphaerales* only contain a few species that are still uncultured and for which limited description is available (Fukunaga et al., 2009). The order *Planctomycetales* is formed by the *Gemmata*, *Blastopirellula*, and *Pirellula* genera, amongst others. The *Cand.* Omnitrophica phylum was recently added to the PVC group with scarce information available, including genomic one.

**FIGURE 2:**
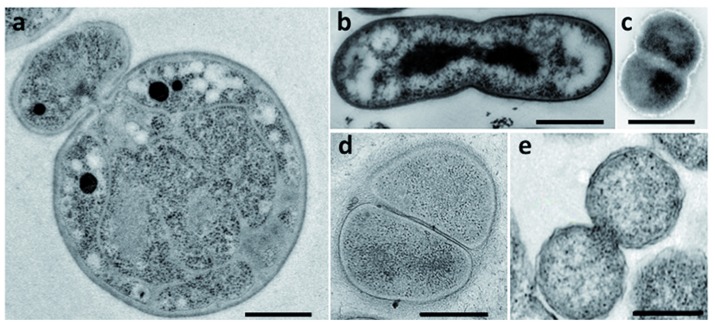
**Division modes in the PVC superphylum.** Transmission electron micrographs of thin sections of dividing cells from **(a)** *G. obscuriglobus* (Santarella-Mellwig, personal communication), **(b)** *Chthoniobacter flavus* [adapted with permission from Sangwan et al. (2004), License number 3662961133966], **(c)** *L. araneosa* [adapted with permission from Cho et al. (2004), License number 3662980203389], **(d)** *P. mikurensis* [reprinted with permission from Fukunaga et al. (2009)], and **(e)** *C. trachomatis* [adapted with permission from Lewis et al. (2014)]. Scale bars, 0.5 μm.

Bacteria belonging to the PVC superphylum are fascinating for their peculiar biology. Their lifestyles range from the free-living soil and aquatic *Planctomycetes*, *Verrucomicrobia*, and *Lentisphaerae*, through the commensal and mutualistic *Verrucomicrobia* and *Lentisphaerae*, to the obligate pathogens of the *Chlamydiae*. *Cand.* Omnitrophica are mostly found in fresh water. Cell division is one of the particularities of this superphylum: some PVC species, such as the members of the order *Planctomycetales*, divide by budding, whereas most others divide by binary fission (**Figure 2**). Although *Chlamydiae* were previously described as dividing by binary fission, new data have revealed an asymmetric division in *Chlamydia trachomatis* (Abdelrahman et al., 2016). With the exception of the *Verrucomicrobia* and *Cand.* Omnitrophica, PVC bacteria also lack the FtsZ protein, the central player of bacterial division. How bacteria deprived of FtsZ divide without this landmark protein is unknown. Some members of the PVC superphylum have also been described as lacking PG. The lack of both PG and FtsZ, and the different mode of division observed in *Planctomycetes* and *Chlamydiae*, once represented one of the biggest mysteries of microbiology – as well as an excellent breeding ground for hypotheses on the evolution of new modes of cell division.

Evolutionary cell biology is a recent field that aims to meld an understanding of evolutionary processes with variation in intracellular structure, based on the comparison of mechanisms between species (Lynch et al., 2014). The development of a new division mode is one of the most important evolutionary transitions. How a binary fission mechanism based on FtsZ evolved into an FtsZ-independent mechanism of division by budding is a major question in this field.

Various publications have recently revealed the presence of PG in some planctomycetal and chlamydial species, suggesting that it is also present in other species in these phyla and in other PVC phyla (Pilhofer et al., 2013; Liechti et al., 2014, 2016; Jeske et al., 2015; van Teeseling et al., 2015). Furthermore, a link between PG synthesis at the septum and division in *Chlamydiae* is emerging, offering clues about the mechanism of bacterial division without FtsZ (Liechti et al., 2016).

Here, we review the most striking features of the PVC bacteria that are linked to cell division without FtsZ and the involvement of PG in this process. We first summarize the historical analyses that led to the conclusion that PG was absent from some of these bacteria. We then recapitulate the recent detection of PG in some chlamydias and planctomycetes, as well as the role of PG, PG-related and division proteins in chlamydial division. Based on the detection of PG-related proteins in PVC proteomes, we evaluate the possible evolution of division mechanisms in these bacteria. Finally, we discuss the implications of these findings for division in *Chlamydiae* and their extrapolation to the other PVC phyla.

### Binary Fission and PG Synthesis

**FIGURE 3:**
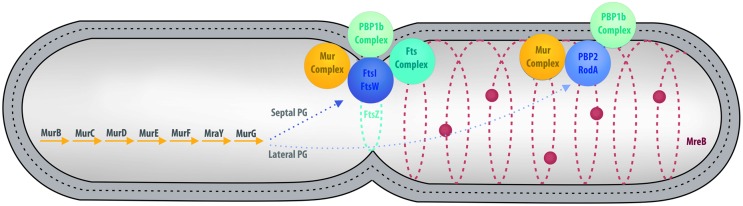
**Overview of cell division in model organisms.** Major cell division and PG synthesis complexes are represented. Cytoplasmic steps of PG synthesis enzymes **(Left)**, major cell division and septal PG synthesis complexes **(Center)**, and elongation of lateral PG complexes **(Right)** are represented by colored spheres. PG is represented by a dotted line between the inner and outer membranes.

In the majority of bacteria, cell division is performed by binary fission. There are a few exceptions: a small proportion of bacteria use mechanisms such as intracellular offspring production or multiple fission (Angert, 2005). Budding is an alternative mode that has been reported in many bacterial lineages, including some members of *Cyanobacteria*, *Firmicutes*, *Planctomycetes*, and the prosthecate proteobacteria. Recent research also reveals that *C. trachomatis* divides asymmetrically, which is reminiscent of budding (Abdelrahman et al., 2016; Liechti et al., 2016). In model organisms, such as *Escherichia coli*, division by binary fission is realized using a molecular machinery assembled around the FtsZ protein, a homolog of the eukaryotic tubulin (**Figure 3**) (den Blaauwen et al., 2008; Erickson et al., 2010). FtsZ is one of the first proteins to localize at the division site in the middle of the cell. It forms a ring while recruiting the components of the division machinery, the divisome, including FtsA, FtsI, FtsK, FtsL, and FtsW, as well as PBP1b, EnvC, NlpD, and the Tol-Pal system (Lutkenhaus et al., 2012). During division, the FtsZ ring and the associated divisome contract, pulling the membranes toward the inside of the cell. As a consequence of its central role in division, FtsZ is present in almost all bacteria. Only a few groups, including *Chlamydiae*, *Planctomycetes*, and some of the *Mollicutes*, are unusual in that they lack a recognizable homolog of FtsZ (**Figure 1**; **Supplementary Table S1**).

Peptidoglycan also plays an important role in division by binary fission. PG is a net-like heteropolymer comprising linear glycan strands that are assembled from two sugar precursors, *N*-acetyl-glucosamine acid (GlcNAc) and *N*-acetyl-muramic acid (MurNAc), through β-(1,4)-glycosidic bonds and stabilized through peptide bridges containing D-amino acids. PG has a structural role: it surrounds the bacterial cell, forming an exoskeleton, the sacculus, that amongst other functions, protects cells from osmotic stress. The sacculus is so stable that it can be purified as a single entity (den Blaauwen et al., 2008). The presence of PG is one of the characteristics that differentiates bacteria from the other two domains of life, archaea and eukaryotes. There are, however, a few exceptions to the PG universality in bacteria, which are now restricted to the *Mollicutes* but until recently also included the *Chlamydiae* and *Planctomycetes*.

### The PVC Melting Pot

Peptidoglycan has historically been thought to be absent from the periplasm of most PVC bacteria. Early attempts failed to detect PG in *Chlamydiae* (Garrett et al., 1974; Fox et al., 1990), efforts to purify chlamydial sacculi were unsuccessful (Caldwell et al., 1981; Barbour et al., 1982), and periplasmic density layers between the inner and outer membranes were not detected by electron microscopy (Tamura et al., 1971; Huang et al., 2010). Surprisingly, bacteria from the *Chlamydiae* are sensitive to β-lactam antibiotics, which inhibit PG synthesis (Matsumoto and Manire, 1970). In addition, most available chlamydial genomes encode functional enzymes for PG synthesis, and the functionality of a nearly complete biosynthesis pathway in several species is well supported (Hesse et al., 2003; McCoy et al., 2003; McCoy and Maurelli, 2005, 2006; Patin et al., 2009, 2012). These contradictory observations have been referred to as the “chlamydial anomaly” (Moulder, 1993). By contrast, *Planctomycetes* are resistant to all PG-targeting antibiotics (Cayrou et al., 2010), and early biochemical analyses were unable to detect PG components in isolated cell envelopes of various strains (König et al., 1984; Stackebrandt et al., 1986). Thus, PG was though to be absent from *Planctomycetes*. Nevertheless, the growth of the anammox planctomycetes *Cand.* Kuenenia stuttgartiensis has been shown to be inhibited by lysozyme and penicillin, which both act on PG (Hu et al., 2013). PG had also been detected in some, but not all, verrucomicrobial species investigated (Yoon et al., 2007a,b, 2008a,b, 2010). In agreement, most PG synthesis genes were detected in *Verrucomicrobium spinosum* and the functionality of at least one of them has been confirmed (McGroty et al., 2013). Recently, PG has been detected in subdivision 5 of *Verrucomicrobia* (Spring et al., 2016).

In addition, and of chief interest here, is the observation that PVC bacteria encode a diversity of division proteins. *Verrucomicrobia*, *Lentisphaera araneosa*, and *Omnitrophica bacterium* OLB16 encode the FtsZ protein and synthesize PG, whereas the *Planctomycetes* and *Chlamydiae* lack FtsZ as well as other division proteins (**Figure 1**; **Supplementary Table S1**). Whereas the *Verrucomicrobia* and *Lentisphaerae*, as well as the lowest branching *Planctomycetes* orders, *Cand.* Brocadiales and *Phycisphaerales*, divide by binary fission, members of the order *Planctomycetales* divide by budding. *Chlamydiae* were previously described as dividing by binary fission, however, asymmetric polarized cell division that might resemble budding has recently been reported in *C. trachomatis* (Abdelrahman et al., 2016; Liechti et al., 2016). This intracellular pathogen has differential localization of major cell components, such as the major outer-membrane protein and lipopolysaccharide present on opposite poles of the cell (Abdelrahman et al., 2016).

Thus, the *Verrucomicrobia* were classified as Gram-negative bacteria dividing by binary fission with FtsZ and PG (Krieg, 2011); the *Chlamydiae* as PG-deprived Gram-negative bacteria dividing by binary fission without FtsZ; and the *Planctomycetes* as dividing by budding without FtsZ and PG, but also as defining a “third cell plan” (Fuerst, 2013b).

### PG Detection in PVC Members

The first analysis to address the conservation of the division and cell wall (*dcw*) cluster in PVC genomes failed to detect some key PG synthesis genes and concluded that the last common ancestor of the PVC superphylum possessed a ‘classical’ *dcw* cluster (Pilhofer et al., 2008). Major modifications, mostly losses, were found in the *Planctomycetes*. The presence of genes was recently re-addressed using a profile-based method more appropriate for detecting remote relationships between proteins (Jeske et al., 2015; van Teeseling et al., 2015). Contrary to previous analyses, these studies revealed that all planctomycetal and chlamydial species investigated harbored the genes essential for PG synthesis.

Given the detection of PG synthesis enzymes in the chlamydial and planctomycetal genomes, the presence of PG in their cell walls was re-evaluated (Pilhofer et al., 2013; Liechti et al., 2014; Jeske et al., 2015; van Teeseling et al., 2015). Using a combination of methods, PG was observed in four planctomycetes strains: *Gemmata obscuriglobus*, *Planctopirus limnophila*, *Rhodopirellula baltica*, and strain L21-Rpul-D3 (Jeske et al., 2015). Those methods included a radioactive kinase assay to detect the transfer of a radioactive γ-phosphoryl group from [γ-^32^P]-ATP to GlcNAc and MurNAc monomers, gas chromatography and mass spectrometry to reveal the presence of the PG component 2,6-diaminopimelic acid, visualization of cell wall sacculi disruption after lysozyme treatment, and direct visualization of a thin PG layer by electron microscopy in vitrified cells. The presence of PG was simultaneously reported for the anammox planctomycete *Cand.* Kuenenia stuttgartiensis (van Teeseling et al., 2015). A similar set of methods was used, including direct observation of sacculi digestion by lysozyme, incorporation of PG specific probes, chemical analyses using ultra-performance liquid chromatography and mass spectrometry, and direct visualization of the cell wall layer by cryo-electron microscopy of vitreous sections. Similarly, the use of mass spectrometry and fluorescent labeling dyes combined with electron cryotomography and a PG synthesis inhibitor revealed the presence of a PG sacculus in *Protochlamydia amoebophila*, a symbiotic member of the *Chlamydiae* (Pilhofer et al., 2013). However, the same technique failed to reveal PG in *Simkania negevensis*, a pathogenic chlamydia-like bacterium. This lack of detection is in agreement with the resistance of this bacterium to penicillin and phosphomycin (Pilhofer et al., 2013), but is at odds with the presence of genes coding for most PG synthesis enzymes in its genome (**Supplementary Table S1**). In addition, two of the *S. negevensis* genes, *amiA* and *nlpD*, have been shown to encode functional proteins that are active in *E. coli* (Frandi et al., 2014). A new metabolic cell-wall labeling method based on D-amino acid dipeptide and click chemistry revealed the presence of PG in *C. trachomatis* (Liechti et al., 2014). Remarkably, whereas a typical, cell-surrounding sacculus is formed in *P. amoebophila*, only a ring-like band is observed at mid-cell in *C. trachomatis* (Liechti et al., 2014).

Individually, these reports provide compelling evidence for the presence of PG in the cell wall of the investigated strains. Taken together, the direct and indirect evidence accumulated from orthogonal sources constitutes irrefutable proof of PG synthesis in phylogenetically dispersed strains of the PVC superphylum. In addition, PG has been detected in strains belonging to the genus Prosthecobacter and in strain L21-Fru-ABT from the *Verrucomicrobia* (Hedlund et al., 1996; Spring et al., 2016). Combined with the genomic data, these detections strongly suggest that PG synthesis takes place in most, if not all, members of the PVC superphylum. To the best of our knowledge, the synthesis of PG has not yet been tested in *Lentisphaerae*, although the only genomic sequence information available so far suggests that *L. araneosa* is capable of PG synthesis (**Figure 1**; **Supplementary Table S1**). These data strongly supports that PVC bacteria are a variation of, not an exception to, the Gram-negative cell plan, putting a clear end to the controversy about the planctomycetal cell plan (Fuerst, 2013b; Devos, 2014a,b; Sagulenko et al., 2014).

### Clarifying the ‘Chlamydial Anomaly’

Considerable modifications of the chlamydial PG structure and timing of its synthesis have recently been revealed in four pathogenic strains: *C. trachomatis*, *C. muridarum*, *Chlamydophila psittaci*, and *C. caviae* (Liechti et al., 2016). The use of chemical probes with super-resolution microscopy demonstrated that PG in these species is limited to a narrow ring at the division plane only during the replicative stage (Liechti et al., 2014, 2016). Because the host immune system recognizes and responds to PG, the authors suggest that members of the family *Chlamydiaceae*, all of which are pathogens, may limit the synthesis of PG to the place and time that are absolutely necessary, i.e., the division septum of replicating cells (Liechti et al., 2016). Thus, PG synthesis is crucial to chlamydial division, even in pathogenic strains, which have reduced it to a minimum. This spatiotemporally limited synthesis of PG explains why it was not detected before and also why *Chlamydiae* are susceptible to antibiotics, thus resolving the ‘chlamydial anomaly.’

### Historical Considerations

The detection of PG in *Planctomycetes* and *Chlamydiae* poses the question of why it remained undetected for so long. In the case of *Chlamydiae*, it was due mostly to technical limitations, combined with the restricted presence of PG in time and space in pathogenic strains (Pilhofer et al., 2013; Liechti et al., 2014, 2016). The case of *Planctomycetes* appears to be different. Since early analyses (König et al., 1984; Liesack et al., 1986; Stackebrandt et al., 1986), the absence of PG in *Planctomycetes* cell walls was not reconsidered, despite several investigations into these bacteria and their structure (Fuerst, 2005, 2013b; Fuerst and Sagulenko, 2013; Sagulenko et al., 2014). Planctomycetal sacculi was purified in the presence of SDS and high temperature, but instead of PG, a proteinaceous cell wall was found (Stackebrandt et al., 1986). However, protein recovery was only 51%, with no indication of what comprised the remaining 49% of the envelope. It is unlikely that the sacculi consisted exclusively of proteins, as proteins of mesophilic organisms cannot resist hours of boiling in SDS and would be denatured under those conditions. Attempts to determine the presence of PG in *Planctomycetes* never involved lysozyme treatment, until recently (Giovannoni et al., 1987; Hu et al., 2013; Jeske et al., 2015; van Teeseling et al., 2015).

Given that PG is present in *Planctomycetes*, their resistance to β-lactam antibiotics might appear confusing (Cayrou et al., 2010). This resistance is possibly due to the presence of putative β-lactamases encoded in their genomes (Kahane et al., 1993). At least one putative β-lactamase gene is present in each *Planctomycetes* genome, often in multiple copies. For instance, there are 13 putative β-lactamases encoded in the *P. limnophila* genome (Labutti et al., 2010). By contrast, *Chlamydiae* are sensitive to β-lactam antibiotics despite the presence of β-lactamases in their genomes (Bertelli et al., 2010).

### Chlamydial Division

The detection of PG in PVC species and its interaction with division proteins are important, as they provide potential clues about PVC division mechanisms. The few PVC species for which division mechanisms have been investigated so far belong to the *Chlamydiae* (Jacquier et al., 2015). Chlamydial division was previously believed to resemble FtsZ-dependent binary fission (Brown and Rockey, 2000; Abdelrahman and Belland, 2005). However, an asymmetric polarized division mode has recently been revealed in *Chlamydiaceae* (Abdelrahman et al., 2016) and progress on understanding the link between chlamydial PG and division has been reported (Jacquier et al., 2015). This new knowledge allows us to draw a preliminary model of chlamydial division that reveals important modifications to the mechanisms of division in model organisms. In particular, the central role of MreB (a bacterial homolog of actin) and its interaction with PG synthesis enzymes at the septum is emerging.

Division is tightly regulated in *Chlamydiales*, as only one specific cell type, the reticulate body, is metabolically active and able to divide. Expression analyses of several division and PG biosynthesis genes in *C. trachomatis* and *C. pneumoniae* show that these genes are over-expressed in the dividing forms compared with the non-dividing forms (Albrecht et al., 2010, 2011). Typical peptide components of PG accumulate at the chlamydial division site, as in most other bacteria (Jacquier et al., 2014). These PG precursors are required for proper localization of PG- and MreB-binding proteins at the division septum (Jacquier et al., 2014). The activity of MurA, MurF, and MurG, which are involved in the early and mid-phase stages of PG synthesis, are required to organize the chlamydial septum, further supporting the understanding that PG synthesis is required for division (Liechti et al., 2014).

The cytoskeletal proteins MreB and RodZ, which are responsible for cell-shape determination in model organisms, seem to play an important role in this process in *Chlamydiae*, as they may bring together the PG biosynthesis and remodeling enzymes to the divisome (Jacquier et al., 2015). Indeed, it appears that FtsZ-less *Chlamydiae* uses MreB to define the division plane by interaction with FtsK, which in turn may recruit PBP2, FtsI (also called PBP3) and likely other unidentified proteins (Ouellette et al., 2012). Co-localization results suggest that MreB polymerization is required to guide new PG incorporation along the PG ring in *Chlamydiales* (Gaballah et al., 2011; Liechti et al., 2016), supporting the proposal that MreB acts as the division plane organizer in pathogenic chlamydias. However, MreB does not seem to be an early cell division protein like FtsZ, because it is recruited late at the septum (Gaballah et al., 2011). Like FtsZ, MreB appears to be a central coordinator of the large multi-protein PG synthesizing complex that cooperates to direct cell elongation. This complex includes PBP2, RodA and many additional partners, such as MreC, MreD, and RodZ, that are involved in the stabilization and/or regulation of this machinery. *C. pneumoniae* MreB has been shown to interact with MurF, MraY, and MurG, three key components of lipid II biosynthesis (Gaballah et al., 2011). In *W. chondrophila*, RodZ is recruited early to the septal site in a process that is dependent on the presence and dispersal of cell wall precursors (Jacquier et al., 2014). The endopeptidase NlpD localizes at the division plane in *Chlamydiae* and its septal sequestration depends on prior cell wall synthesis (Frandi et al., 2014). Thus, the division mechanisms of *Chlamydiae* share similarities but also have important differences with model organisms.

### Evolutionary Cell Biology of Division Modes in PVC Bacteria

The diversity of characteristics of related bacteria makes the PVC superphylum particularly attractive for the field of evolutionary cell biology. To paraphrase Theodosius Dobzhansky, it could be said that “nothing in PVC cell division makes sense except in the light of evolutionary cell biology.” Some clues about PVC division can be derived from the limited knowledge available about division in the *Chlamydiae* and from gene conservation in other species. We thus revisited the presence of the main genes involved in PG synthesis and division in the PVC proteomes for a representative set of genomes across the superphylum (**Figure 1**; **Supplementary Table S1**). It is unclear if the inability to detect some proteins is due to their absence or represents a false negative result. The latter nevertheless indicates a profound modification of the protein sequences and thus most likely of the molecular mechanisms associated with it.

**FIGURE 4:**
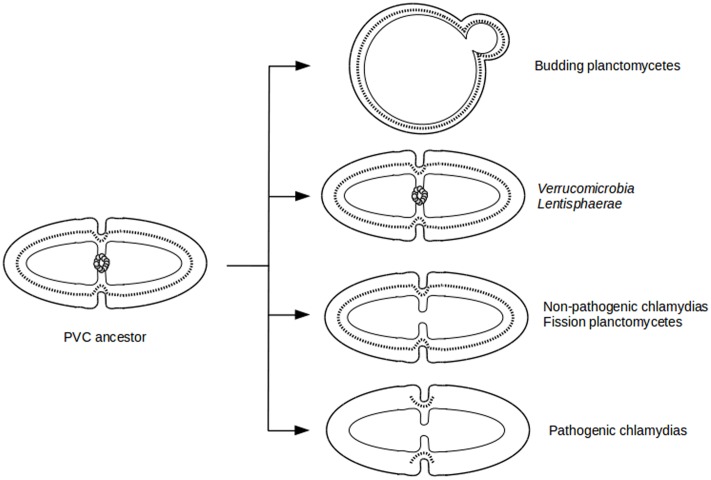
**Evolutionary cell biology of division modes in PVCs.** Schematic representation of cell division modes in the last PVC common ancestor **(Left)** and in current PVC species **(Right)**. Outer and inner membranes are in thick and thin lines, respectively. PG layer is in dotted line. FtsZ proteins are represented as a ring of gray spheres.

The diversity of division modes and proteins observed in PVC species is due to divergent evolution from a common ancestor (**Figure 4**). The most parsimonious explanation for the gene distribution of the *dcw* cluster is that the last PVC common ancestor was a Gram-negative bacterium that divided by binary fission using PG and FtsZ, and that had a mostly classical *dcw* cluster, as suggested by others (Pilhofer et al., 2008; Jogler et al., 2012). Within the PVC superphylum, species have diverged from this common ancestor, accumulating differences including gene losses but also modifications of the function of existing genes (**Figure 1**; **Supplementary Table S1**). How gene losses are related to modifications of this division mechanism is one of the main questions remaining in this field. Even in the PVCs containing FtsZ, modifications of the division modes are to be expected, as demonstrated by the losses of molecular components. Due to the diversity of phenotypes and genotypes encountered in the PVC superphylum, generalization of their features is a difficult exercise.

First and foremost, FtsZ, the landmark protein of binary fission that is conserved in almost all bacteria, is not found in *Chlamydiae* or in *Planctomycetes*. It is detected only in *Omnitrophica bacterium* OLB16, and it is not clear if its absence in the other two members of the *Cand.* Omnitrophica is real or merely a result of incomplete data. On the other side of the spectrum of conservation, a putative FtsK protein, the DNA translocase, is likely to be present in all genomes (its absence in *Zavarzinella formosa* is likely due to incomplete data). The same applies to MraY, an integral membrane enzyme that catalyzes the transfer of the PG precursor to the lipid carrier undecaprenyl phosphate, forming the lipid I.

In between those two extremes, interesting intermediary cases are found. MreB is found in almost all PVC proteomes, with the exception of some of the budding planctomycetes. This conservation supports the central role of MreB in the absence of FtsZ. In model organisms, MreB requires the membrane proteins MreC and MreD for the organization of the PG (White et al., 2010). Interestingly, MreD is not found in any PVC proteomes, while MreC shows a patchy distribution: it is found in verrucomicrobias and chlamydias, and in two out of three binary fission planctomycetes, but not in *L. araneosa* or in the budding planctomycetes (with the possible exception of *Planctomyces brasiliensis*, which is probably a false positive*)*. The protein RodZ might form the link between MreB and the PG-modifying penicillin-binding proteins (PBPs), and it locates early at the septum in *Chlamydiae* (Jacquier et al., 2014). Interestingly, RodZ is only found in *Chlamydiae* and *Verrucomicrobia* but not in *Planctomycetes* or in *L. araneosa*. Thus, important modifications of division mode due to the lack of RodZ are expected. This is in agreement with the fact that the chlamydial RodZ is truncated, lacking its C-terminal periplasmic domain, and cannot complement an *E. coli* RodZ mutant (Ouellette et al., 2014).

The peptidase NlpD is another protein that localizes at the septum in chlamydial pathogens (Frandi et al., 2014). NlpD, like RodZ, is found only in *Chlamydiae* and *Verrucomicrobia*, and not in *Planctomycetes* or *Lentisphaera*. The amidase activator EnvC is not found in any PVC proteomes (with a single exception, which is probably a false positive). In model organisms, EnvC is a regulator of the cell wall amidases AmiA and AmiB. Like NlpD, AmiA is found in *Chlamydiae* and *Verrucomicrobia*, but not in *Planctomycetes, Omnitrophica*, or *Lentisphaera*. AmiB is detected in *Chlamydiae*, *L. araneosa*, and only one *Omnitrophica*, but only in five members of the *Verrucomicrobia* (out of nine) and not at all in *Planctomycetes*. Most PG synthesis enzymes are found in PVC members, with the exception of some budding planctomycetes.

Hence, this pattern of undetected proteins illustrates important modifications of the cell division modes in the various branches of the PVC superphylum. The diverse division modes in PVC bacteria offer a fascinating field of study for the years to come.

## Remarks And Open Questions

Great progress has been made in uncovering the division modes in members of the PVC superphylum. Many relevant discoveries reveal new questions that require further research to answer. The data presented here calls for a few remarks and questions.

First, PG is almost universal in bacteria, with a few exceptions limited to obligate intracellular bacteria, and is one of the true features defining bacteria (including bacteria-derived organelles such as plastids). The recent detection of PG in PVC members, combined with genomic information, suggests that it is present in most PVC species. The only bacterial group that is possibly devoid of a PG cell wall is the *Mollicutes*. In addition, to the best of our knowledge, the mollicutes *Phytoplasma* sp., *Ureaplasma* sp., and *Mycoplasma mobile* are the only species deprived of both FtsZ and PG (Bernander and Ettema, 2010).

Second, PVC bacteria are variations of, but not exceptions to, the Gram-negative cell plan (Devos, 2014a). The presence of PG in PVC species definitively resolves this controversy.

Third, most PVC bacteria have a well developed endomembrane system. This system can take the form of invaginations of the cytoplasmic membrane toward the inside of the cell (as observed in most planctomycetes), of evaginations of the outer membrane (in some verrucomicrobias), or of a tubulo-vesicular network in the periplasm of the cell (in the planctomycete *G. obscuriglobus*; Santarella-Mellwig et al., 2013; Acehan et al., 2014; Devos, 2014a). The anammox planctomycetes even appear to display the first prokaryotic organelle with a compartment apparently separated from the inner membrane (Neumann et al., 2014). How the PG organizes within or around this endomembrane system, including the periplasmic tubulo-vesicular network, is another intriguing feature that requires deeper investigation. How PG is synthesized and organized in the bud is yet another interesting area of research.

Fourth, during evolutionary divergence from their common ancestor with a classical FtsZ-based binary fission mode of division, PVC members have evolved different modifications, including the loss of FtsZ and the development of alternative division mechanisms and modes, raising various questions for evolutionary cell biology. What forces and constraints acted on which proteins and processes? What has been the evolutionary pressure behind those innovations? Given the degradation of the *dcw* cluster, the total conservation of some genes, such as *mreB* or *ftsK*, is intriguing.

Fifth, recent progress has been made in deciphering the chlamydial division mechanism, which requires the active synthesis of PG, suggesting a role at the septum related to that of PG in classical Gram-negative species. This opens up more important questions. How much of the ancestral division mechanism is conserved in current species? And what are the differences? What is the role of PG in division without FtsZ? Has MreB replaced FtsZ, as has been suggested (Jacquier et al., 2015)? A key question is how to transfer the chlamydial knowledge to other PVCs.

Sixth, recent advances in the field of PVC genetic manipulation are providing us with better tools to answer some of these questions. Genetic tools have been described recently in the three initial PVC phyla *Planctomycetes*, *Verrucomicrobia*, and *Chlamydiae* (Domman et al., 2011; Jogler et al., 2011; Kari et al., 2011; Wang et al., 2011; Gérard et al., 2013; Erbilgin et al., 2014; Kokes et al., 2015; Rivas-Marín et al., 2016). The analyses reported here and elsewhere constitute a very good place not only to continue deciphering the modes of division of *Chlamydiae*, but also to start exploring those of the other PVC members. The isolation and characterization of new PVC members is important, as it will provide more information about the diversity of division modes in PVC bacteria. Is there an intermediary organism with a mode of division between binary fission and budding? Genome information for additional PVC species is needed to evaluate the conservation of division proteins (and mechanisms) in this superphylum.

## Conclusion

The PVC bacterial superphylum presents an unparalleled opportunity for the study of evolutionary cell biology of division modes. We are looking forward to progress in this fascinating field.

## Author Contributions

All authors listed, have made substantial, direct and intellectual contribution to the work, and approved it for publication.

## Conflict of Interest Statement

The authors declare that the research was conducted in the absence of any commercial or financial relationships that could be construed as a potential conflict of interest.

## Supplementary Material

TABLE S1 **Division and cell wall cluster and PG synthesis protein detection in PVC**. Due to the divergence of the PVC species from model organisms and for the sake of completeness, two complementary searches were combined (Sheet 1). We first performed two iterations of Psi-Blast using the *W. chondrophila* (Sheet 2) and *E. coli* (Sheet 3) proteins as a query on the non-redundant GenBank (nr) database, using default parameters and accepting hits with an *e*-value below 10^-3^. One additional iteration was run on the PVC proteomes with the obtained profile as query. A reciprocal search was then used to verify that the starting proteins were found when using the hit as a query. The PVC hit was only accepted if the reciprocal search found the starting sequence. Each cell contains two identifiers, if the reciprocal best hits are different, the first one corresponding to the hit from the *W. chondrophila* search (left), followed by the hit from the *E. coli* search (right). In most cases, the two approaches hit the same proteins, reinforcing their results. Only one identifier is indicated if the reciprocal best hits are the sames. Sheets 2 and 3 show the *e*-values of detection corresponding to the searches from the *W. chondrophila* and *E. coli* proteins, respectively. NA, not applicable; PF, binary fission planctomycetes; PB, budding planctomycetes.
